# A Systematic Review of Occupational Exposure to Particulate Matter and Cardiovascular Disease

**DOI:** 10.3390/ijerph7041773

**Published:** 2010-04-19

**Authors:** Shona C. Fang, Adrian Cassidy, David C. Christiani

**Affiliations:** 1 Department of Environmental Health, Harvard School of Public Health, Boston, MA 02115, USA; 2 Epidemiology Unit, Health and Safety Executive, Bootle, L20 7HS, UK; E-Mail: cassidya@liv.ac.uk; 3 Department of Medicine, Massachusetts General Hospital/Harvard Medical School, Boston, MA 02114, USA; E-Mail: dchris@hsph.harvard.edu

**Keywords:** particles, air pollution, epidemiology, environmental health, ischemic heart disease, occupation, inflammation, heart rate variability, meta-analysis

## Abstract

Exposure to ambient particulate air pollution is a recognized risk factor for cardiovascular disease; however the link between occupational particulate exposures and adverse cardiovascular events is less clear. We conducted a systematic review, including meta-analysis where appropriate, of the epidemiologic association between occupational exposure to particulate matter and cardiovascular disease. Out of 697 articles meeting our initial criteria, 37 articles published from January 1990 to April 2009 (12 mortality; 5 morbidity; and 20 intermediate cardiovascular endpoints) were included. Results suggest a possible association between occupational particulate exposures and ischemic heart disease (IHD) mortality as well as non-fatal myocardial infarction (MI), and stronger evidence of associations with heart rate variability and systemic inflammation, potential intermediates between occupational PM exposure and IHD. In meta-analysis of mortality studies, a significant increase in IHD was observed (meta-IRR = 1.16; 95% CI: 1.06–1.26), however these data were limited by lack of adequate control for smoking and other potential confounders. Further research is needed to better clarify the magnitude of the potential risk of the development and aggravation of IHD associated with short and long-term occupational particulate exposures and to clarify the clinical significance of acute and chronic changes in intermediate cardiovascular outcomes.

## 1. Introduction

Cardiovascular disease (CVD) is the leading cause of death and morbidity worldwide [1], and within recent years, research studies have increasingly sought to understand the environmental determinants. In particular, much attention has focused on ambient particulate air pollution. As reviewed in several places [2–4], exposure to particulate matter (PM) as a result of air pollution has become a recognized risk factor for adverse cardiovascular events including cardiovascular mortality, cardiac arrhythmia, myocardial infarction (MI), myocardial ischemia, and heart failure [5–12]. The elderly and those with pre-existing cardiovascular disease appear to be especially vulnerable [2]. Estimates place close to one million individuals at risk of death from CVD related to particulate air pollution exposures (*i.e*., not including estimates of morbidity) worldwide each year [13]. With regard to non-fatal outcomes, the risk of MI has been estimated to be 1.48 times greater (95% CI: 1.09–2.02) for a small increase in particulate air pollution (25 μg/m^3^ in the preceding two hours) [5]. In comparison, the risk of MI was recently reported to be nearly 3 times higher in current smokers as compared to never smokers (95% CI: 2.77–3.14) [14]. While the increase in risk of adverse clinical cardiovascular outcomes associated with particulate air pollution is relatively small compared to traditional risk factors such as smoking, as well as diet, obesity, diabetes, metabolic syndrome, *etc.* the ubiquity of particulate air pollution exposes a much larger number of individuals in the population and over an entire lifetime. Thus the relatively small cardiovascular effects of PM translate into a serious and large public health impact.

The smaller sized particles, that is, fine (PM_2.5_) and ultrafine particles (UFP) (particulate matter with a mass median aerodynamic diameter ≤ 2.5 μm and 0.1 μm, respectively), primarily produced from combustion sources, have been shown to be especially toxic [9,15,16]. While the precise biological mechanisms linking PM exposure with CVD is yet unclear, likely mechanisms include systemic inflammation subsequent to pulmonary inflammation, alterations of the autonomic nervous system that lead to changes in heart rate and heart rate variability, and translocation of particles (specifically UFP) or soluble components (e.g., transition metals) from the lungs directly into the systemic circulation [17].

Despite a large body of evidence of the deleterious effects of ambient PM exposures on the cardiovascular system, relatively little attention has been given to the effects of occupational exposures, which differ from general ambient exposures in both particle type (e.g., composition), as well as exposure frequency (e.g., environmental exposures are relatively constant while occupational exposures are more variable), duration (e.g., a work-shift and working lifetime *vs.* an entire day and lifetime), and intensity or concentration (*i.e*., occupational exposures are generally higher than ambient levels). These differences in exposure composition, duration, frequency and population exposed may have implications on how occupational PM exposures impact CVD and how these associations are studied. For example, intense exposures may act as an acute trigger of acute coronary events for individuals with pre-existing cardiovascular disease. At the same time, differences between the general population and working populations may also have implications on how particulate exposures impact workers’ cardiovascular health. For example, working individuals are healthier than the general population and may be less responsive to particulate exposures, but over a working lifetime chronic insults may lead to pathophysiologic responses. Globally, millions of workers are occupationally exposed to PM, and the regular, frequent, and prolonged exposure may increase the risk of adverse cardiovascular health effects in exposed workers.

The relatively few published studies that address the association between occupational PM exposure and CVD vary greatly in terms of the specific types of PM exposures, confounding co-pollutants present, and outcomes investigated. To date, one 1997 narrative review of the association between occupational exposure to PM and CVD has been published [18] which focused on case-control studies of a wide variety of PM exposures (quartz, asbestos, PAH, arsenic, beryllium, and lead, and work as farmers, pulp and paper workers, sawyers, and welders) in relation to ischemic heart disease (IHD), citing some evidence of an association. However, to our knowledge, a formal systematic review has not been performed. For this reason, we conducted a systematic review, including meta-analysis where appropriate, of the epidemiologic evidence on the association between occupational particulate exposures and CVD. As the types of PM exposures in the workplace are historically and geographically varied, this review focused on exposures germane to modern industrialized countries and was limited to solid non-fibrous particles found in inorganic dusts and fumes. In addition, this restriction on particle type was chosen because although the potential cardiovascular effects and mediating mechanisms may vary within this broad category of PM, they are likely to be more similar than the effects of fibers (e.g., asbestos fibers) and organic dusts (e.g., wood dust and other vegetable dusts), the latter which generally contain endotoxin, a known potent inflammatory stimulus [19].

## 2. Methods

### 2.1. Search Strategy

Observational studies investigating the association between particulate exposures in the workplace and cardiovascular outcomes, including both clinical (fatal and non-fatal) and intermediate outcomes were identified in PubMed (www.ncbi.nlm.nih.gov/pubmed) and EMBASE (www.embase.com) by searching free text and key words (Appendix). Search terms for occupational particulate exposures included “particulate matter” as well as specific types of PM exposures (silica, styrene, diesel exhaust, asphalt fumes, and metal or welding fumes), which contain particles in the fine or ultrafine size range [20–24]. The literature search was restricted to articles published in the English language from January 1, 1990 through April 2009. We also used secondary references cited by the articles identified in the primary search.

Because few mortality studies specifically investigated occupational PM exposures and CVD mortality, we chose to include general all-cause mortality studies where CVD was among the cause-specific associations presented. However, we excluded mortality studies that explicitly stated that the objective was to investigate an outcome other than CVD (e.g., cancer or respiratory disease) because not all such studies presented CVD mortality in findings. Hence, even if CVD mortality were presented in the findings, such studies were excluded. In addition, we excluded studies set in industries or among occupations for which PM was not the exposure of interest, or in which exposure to PM was not the predominant exposure. We also excluded publications that did not contain original research (e.g., reviews, editorials, and letters), studies not carried out in humans (e.g., animal and other experimental studies), and case reports and case series.

Additionally, for studies conducted in the same cohort with the same outcome and exposure, we chose the cohort with largest sample size. We included studies that re-analyzed data in the same cohort to identify specific components of PM responsible for health effects and also included studies that aimed to identify effect modifiers.

### 2.2. Data Extraction

For each study that met the study criteria, we extracted information on the study characteristics (authors, year of publication, country, study design), population characteristics (inclusion criteria, age, industry, gender), exposure assessment, outcome ascertainment, and measures of association. Measures of association extracted or derived from the published data were standardized mortality ratios (SMR), incidence rate ratios (IRR), odds ratios (OR), proportional mortality ratios (PMR), regression coefficients (ß), and their standard errors. Where there were more than one analytical comparison group (*i.e*., external and internal), both measures of association were extracted. For mortality studies, data were extracted for the major categories of IHD (ICD–9 410–414), cerebrovascular disease (ICD–9 430–438), and overall CVD (ICD–9 390–459) when presented. For morbidity studies, we extracted data on any cardiovascular outcome presented, and for intermediate outcomes we investigated measures including systemic inflammation, blood pressure, cardiac abnormalities, heart rate and heart rate variability. Additional study characteristics, which may be used to assess the strengths and weaknesses of each of the mortality studies, were extracted and are included in the Appendix.

### 2.3. Statistical Methods

For mortality outcomes, pooled estimates of the SMR and IRR, “meta-SMR” and “meta-IRR”, respectively, with associated 95% confidence intervals were obtained when the study designs and contrasts were comparable. Formal tests for heterogeneity were conducted; however all meta-analyses were performed using random effects meta-analysis of the natural log transformed effect estimates regardless of the test result. For each of the major cardiovascular mortality outcomes, sub-group analyses were performed by type of particulate exposure where possible (SMR studies only). All analyses were performed with STATA Version 10.1 (College Station, TX, US). Meta-analyses were not performed for the morbidity studies or studies of intermediate outcomes because of the non-comparability in study designs.

Because of the small number of studies available for meta-analyses, no further analyses were performed to assess the influence of individual studies on the overall pooled estimates, from either the main analyses or sub-group analyses. Also because of the small number of studies, publication bias was not assessed formally.

## 3. Results

### 3.1. Literature Search

**Figure 1.**
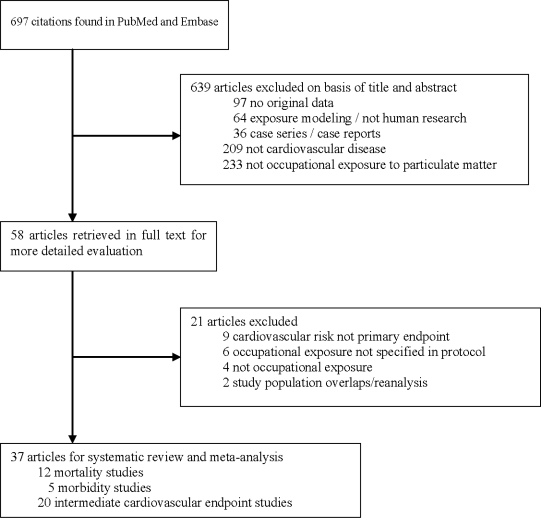
Selection process for identifying original articles for the systematic review of the association between occupational exposure to particulate matter and cardiovascular disease.

Figure 1 presents the selection process for identifying original articles for the systematic review of occupational particulate exposures and CVD. The literature search identified 697 citations (Figure 1), of which a large majority was excluded based on review of the title and abstract. The full text of 58 articles were retrieved and reviewed, and 21 articles were subsequently excluded, including 6 studies of particulate exposures not considered to be germane to this review. A total of 37 articles were identified for review: 12 mortality studies; 5 studies considering morbidity due to CVD, two of which also included mortality [25,26]; and 20 studies concerning intermediate cardiovascular outcomes in relation to acute (17 studies) and/or chronic exposure (4 studies).

## 4. Occupational Particulate Exposures and Cardiovascular Disease Mortality

### 4.1. Study Characteristics

Study characteristics from each of the twelve mortality studies are presented in Table 1. All but two of the mortality studies [27,28] had a specific objective to study a cardiovascular endpoint. The studies were conducted in a large range of industries and occupations, such as gold mining, trucking, and synthetic rubber industry workers, and others. Mortality due to overall CVD was assessed in seven studies [27,29–34], IHD in ten studies [28,30–33,35–38], and cerebrovascular disease in five studies [29,30,31,35,38] (Table 1). The sample sizes were large, ranging from 3,431 to 176,309 workers, excluding external and internal control groups, and were primarily male. Almost all studies used an external reference group for statistical comparison (Table 2), though some used an internal reference only [32,33,38] (Table 3), and some used both [28,29,34,35,37] (Table 3). Half of the studies estimated exposure [30,32,33,35,37,38] however few actually presented estimates of exposure [32,35,37]. Exposure-response relationships using continuous exposure was presented in only one study [35]. Most studies obtained cause of death from death certificates [27,30,31,33,36–38] (Appendix) and most coding was performed with the International Classification of Disease (ICD) system, with the exception of a study of ceramics workers in China exposed to silica dust [29]. Additional study characteristics are presented in a table in the Appendix.

### 4.2. Effect Estimates and Meta-Analyses of Mortality Studies Using External Comparisons

Studies that used external comparison groups standardized the effect estimates (SMRs) to typical population characteristics such as age and calendar year and in some cases race. Effect estimates varied widely across the cohorts and by major outcome (Tables 2). The SMR ranged from 0.90 [37] to 1.41 [31] for IHD; 0.69 [31] to 1.09 [35] for cerebrovascular disease; and 0.73 [31] to 1.06 [29] for overall CVD, with some statistically significant positive associations observed for IHD [31,35,36], none for cerebrovascular disease, and none for CVD. Significantly increased risk of IHD mortality was observed in miners and other workers exposed to silica and trucking industry workers exposed to diesel exhaust particles [31,35,36]. While studies that used an external comparison groups could not control for confounding by co-exposures or other risk factors for CVD such as smoking, one study of asphalt workers, in which a non-statistically significant increase in risk of IHD was found, compared smoking rates in a subset of the study population finding that they were similar to rates in the general male population, suggesting that confounding by smoking habits was minimal [30].

**Figure 2.**
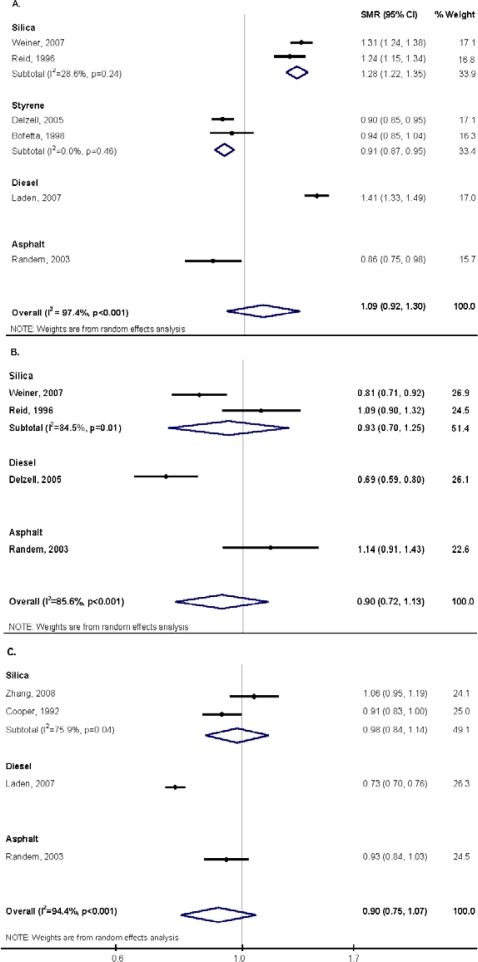
Random effects meta-analysis of mortality due to (A) ischemic heart disease, (B) cerebrovascular disease; and (C) overall cardiovascular disease associated with occupational exposure to particulate matter and by type of particle. Effects estimates are standardized mortality ratios (SMR) based on comparisons with external reference groups.

The SMR from seven studies that presented findings for IHD were combined [28,30,31,35–37], giving a slight but non-significant, excess in deaths due to IHD (meta-SMR = 1.09; 95% CI: 0.92, 1.30) (Figure 2). No excess in deaths due to cerebrovascular disease were found when the SMRs from four studies were combined [30,35–37] (Figure 2) or for overall CVD when the SMRs from four studies were combined [27,29–31] (Figure 2). There was statistically significant (p < 0.05) heterogeneity between studies in the three meta-analyses.

In sub-group meta-analyses, we were able to perform analyses for silica and styrene only (Figure 2). Excess deaths due to IHD mortality were observed in the silica-exposed cohorts [35,36] while a decreased risk of IHD mortality was found in the styrene exposed cohorts [28,37]. No associations were observed for silica and cerebrovascular disease or overall cardiovascular disease in the sub-group meta-analyses.

### 4.3. Effect Estimates and Meta-Analyses of Mortality Studies Using Internal Comparisons

#### Ischemic Heart Disease

Six studies investigated the association between increasing occupational PM exposure and IHD using internal analyses [32,33,35,37,38] (Table 3). The comparisons used in the analyses varied, precluding a direct comparison of all the IRRs, which ranged from 0.90 to 1.64. While most studies compared IHD mortality in high or medium exposed workers to low or unexposed workers, different exposure metrics were used (e.g., cumulative exposure, lifetime average intensity, average exposure) [32–34,37]. The other studies compared a unit increase in cumulative exposure [35], any *versus* no exposure [38], and job title [34]. In most of these studies, positive effect estimates for IHD were observed, though not all were statistically significant, and adjustment for smoking [35,38] and other important potential confounders, such as co-pollutants [37], was limited.

In a large study of construction workers by Toren *et al*. a significantly increased risk of IHD was observed (IRR = 1.12; 95% CI: 1.10–1.14) when those with occupational exposure to any dust were compared to those with no exposure [38]. When evaluated by type of particulate exposure, increased risk of IHD was observed in association with inorganic dust, fumes, and diesel exhaust particles (Table 3). The authors controlled for baseline category of smoking, age and baseline body mass index, potentially important confounders. Time-varying data on exposure was not available, as well as smoking and body mass index, which may co-vary with exposure over time. However, the authors reported that job mobility was limited during the study period and that exposure levels remained high at the end of the study period, and thus the lack of time-varying exposure data should not have been a major limitation. While the authors found similar results when restricting to baseline never smokers, this does not rule out uncontrolled confounding by changes in smoking patterns over time which could have biased the effects in either direction. It is also possible that the associations were partially due to increasing body mass index over time if this were correlated with exposure, but changes in BMI are unlikely to account for all of the observed effects since the authors noted that the exposures remained steady at the end of the study period. The authors also controlled for hypertension as controls had a slightly lower rate of hypertension than the exposed population. However hypertension, may be an intermediate between occupational PM exposure and IHD [39], and controlling for it in the model may attenuate the potential association between occupational PM exposure and IHD, biasing the effects towards the null. An analysis stratified by hypertension would have been more useful.

Significant increases in risk of IHD were also observed in heavy equipment operators compared to other unionized workers (OR = 1.23; 95%CI: 1.00–1.51) controlling for age [34], metal workers exposed to high levels of dust compared to low exposed workers (IRR = 1.40; 95% CI: 1.19–1.74) controlling for age, and asphalt workers exposed to high average coal tar exposures compared to low exposed workers (IRR = 1.64; 95% CI: 1.13–2.38) [32] (Table 3). No adjustment for smoking was performed, but in the study of asphalt workers, the authors conducted a sensitivity analysis utilizing the distribution of smokers in a subset of the population, concluding that confounding by smoking was not a major limitation. The authors did acknowledge however that the increased risk of IHD could be due to polycyclic aromatic hydrocarbons (PAH) or carbon monoxide (CO) exposures from other occupations outside of asphalt work, but this could not be determined because of lack of complete occupational histories [32].

In a study of gold miners exposed to silica dust, in which cumulative exposure was treated as a continuous variable, [35] no associations were observed with IHD while adjusting for smoking patterns. Notably, in this same study, a significant SMR for IHD was obtained when using the general population as a reference group. These contradictory findings may be explained by the fact that exposure was lagged by five years in the cumulative exposure models to account for the latent period between exposure and disease. If more recent PM exposures were critical in the onset of IHD or triggering of an acute event, then lagging the exposure would have masked an effect of PM. This study also adjusted for blood pressure, which may be an intermediate outcome on the pathway between PM exposure and IHD. Another study investigated risk of IHD in plastics industry workers exposed to styrene particles finding no association when comparing category of average intensity and cumulative exposure, but the authors noted that exposures estimates were not validated and exposure misclassification was likely. In addition, the authors did not control for smoking or other potential confounders [37]. In contrast to the null IRR, the SMR from this study was significantly lower than unity, suggesting a protective association, which may reflect a bias from the healthy worker effect in the external analysis.

**Figure 3.**
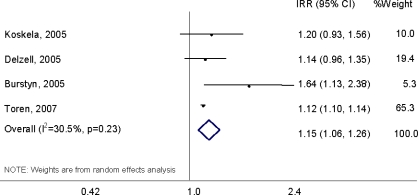
Results of random-effects meta-analysis of IHD mortality using internal analysis and occupational exposure to particulate matter.

When the IRRs from the four studies that compared high or any verses low or no exposure [32,33,37,38] were combined in a meta-analysis, there was a 15% increased risk of IHD mortality (meta-IRR = 1.15; 95% CI: 1.06–1.26) (Figure 3). While the result suggests a significantly increased risk of IHD mortality with higher exposure, control for confounding factors was limited in the original studies, with only one study having controlled for smoking [38]. No significant heterogeneity between studies was observed. Fixed-effects meta-analysis did not alter the meta-IRR for IHD appreciably (meta-IRR = 1.12; 95% CI: 1.10–1.14). Meta-analyses by type of particle exposure could not be performed for studies using internal analyses because of lack of comparability in the study designs by exposure.

#### Cerebrovascular Disease

Only one study investigated cerebrovascular mortality using an internal comparison finding no association with any dust exposure [38]. This was investigated by Toren *et al*. in the study of construction workers. Since the authors controlled for blood pressure, it is possible that an effect was masked by controlling for an intermediate variable.

#### Overall Cardiovascular Disease

Three studies investigated overall CVD mortality using internal analyses. Mortality was compared by categories of exposure, as measured by cumulative exposure and average exposure and findings were mixed with positive and inverse association. The IRRs varied from 0.77 [29] to 1.85 [32] comparing high to low exposure. Bustyn *et al*. reported a significant increase in CVD mortality in asphalt fume workers exposed to high average coal tar (IRR = 1.85; 95% CI: 1.17–2.91) [32]. However, as with their analysis for IHD mortality, adjustment for confounding was not performed so it is possible that the increase in risk was due to exposure to PAHs or CO from other occupations. In the study by Koskela *et al*. an increased risk of overall CVD was observed for metals workers with high exposure compared to those with low exposure (IRR = 1.20; 95% CI: 1.04–1.35), adjusting for age, but not smoking or other risk factors. Zhang *et al*. found a significantly decreased risk of overall CVD in ceramics workers exposed to high cumulative exposures as compared to low exposed workers in crude analysis [29]. Notably, coding of cause of death was not based on the ICD system and thus it is difficult to compare the findings from this study to others. In addition, the apparently protective effect of exposure may reflect a healthy worker survivor effect, in which healthier workers are able to withstand work in dustier jobs. No additional analyses were conducted to control for potential confounders. When the IRRs from these three studies were combined, the meta-IRR for CVD mortality was 1.17 (95% CI: 0.50 to 2.75) for those with high exposure *versus* low exposure (not presented). Significant heterogeneity between studies was observed in the CVD meta-analysis. Meta-analyses by type of particle exposure could not be performed for studies using internal analyses because of lack of comparability in the study designs by exposure.

In summary, a number of positive associations with IHD mortality were observed in the studies that used external comparison groups. While these studies are limited by the lack of control for important cofounders such as smoking and co-pollutants, the lack of a healthy worker effect bias which typically manifests as a protective association for IHD argues for a potentially real association between occupational PM exposure and IHD. In fact, only one study of styrene exposed workers observed a significantly reduced SMR [37].

Using an internal comparison group helps to minimize the healthy worker effect and is preferential over the use of external comparisons for this reason. Findings from the mortality studies that used internal analyses also revealed positive associations with IHD. While confounding by lifestyle and demographic habits is minimized in internal analyses where the population is more similar, the findings here are still limited by lack of adequate control for confounding in most studies. Only one study with a positive association between any occupational exposure to PM among construction workers and IHD controlled for smoking and age in the analysis [38].

## 5. Occupational Particulate Exposures and Cardiovascular Morbidity

### 5.1. Study Characteristics

The study characteristics and major results of the five morbidity studies are presented in Table 4. All but one study, which evaluated IHD and overall CVD prevalence [40], specifically assessed incidence or prevalence of MI [25,26,41,42]. The study populations were also relatively large, ranging from 2,993 to 153,807 individuals and mostly male. All of the study populations were drawn from the general population. In general, hospital data were used to ascertain outcome. Job title [25,40,42] and exposure estimates [26,41] (lifetime average intensity and duration) were used to assign exposure. Several studies adjusted for smoking as a confounding factor of cardiovascular morbidity [40,41]. One study collected information on smoking but it was unclear if this was adjusted for in the analysis [26]. In the study of occupations exposed to traffic-related particles [25], the authors indirectly evaluated the impact of smoking and body mass index on the association between occupations exposed to PM and MI using varying distributions.

### 5.2. Effect Estimates

In a cross-sectional study, IHD and CVD prevalence rate ratios among transport workers compared to the general employed population were below unity and null [40]. While smoking and age were appropriately controlled for, the study included individuals as young as 16 years of age, who would be less likely to have any type of CVD. In another cross-sectional analysis, the prevalence of MI was found to be increased in individuals with long-term exposure (greater than five years) to welding and soldering fumes compared to those without long-term exposure. However smoking and other potential confounders did not appear to be controlled for in the analysis, although the authors did report collecting information on smoking [26]. In this same study, the incidence of MI was also studied prospectively finding an elevated risk of MI in individuals exposed to soldering fumes long-term, but not those exposed to welding fumes. Again it was unclear whether potential confounders were controlled for in the analysis.

In the three case-control studies that investigated the incidence of MI, positive effect estimates were observed in almost all seven comparisons that were included in this review though not all associations were statistically significant (Table 4). In a study of urban males employed in occupations exposed to traffic-related particles, statistically significant effect estimates for risk of MI ranged from 1.31 to 1.53 for bus drivers, taxi drivers and long distance lorry drivers as compared to gainfully employed men in the general population while controlling for age and other potential confounders. Smoking and BMI were not adjusted for, but the authors were able to indirectly evaluate the impact of these factors, finding that smoking and obesity were unlikely to account for all of the increased risk in MI, especially in taxi and bus drivers.

In a subsequent study by the same authors [41], male and female workers exposed to high levels of respirable combustion-related PM as determined from a lifetime occupational history questionnaire experienced a more than two-fold increased risk of MI compared to unexposed individuals, while controlling for smoking and a number of other covariates, including age, diabetes and overweight. The authors did control for hypertension however, a factor which may be on the causal pathway between exposure and MI and which therefore may attenuate the effect estimate when included in the model. In the third case-control study by Bigert et *al*. [42] of subway drivers exposed to underground particulate matter, no increased risk of MI was found when workers were compared to gainfully employed men in the general population or other manual workers. The authors were only able to control for age and calendar year in this analysis.

Control for confounding factors and use of appropriate comparison groups (*i.e*., gainfully employed individuals as opposed to the general population) was in general better in these morbidity studies. However the lack of information on co-exposures such as CO and direct measurements of particulate exposures to estimate long-term exposure and evaluate exposure-response relationships remain as important limitations to these studies, which otherwise suggest an association between occupational PM exposure and MI.

## 6. Occupational Particulate Exposures and Intermediate Cardiovascular Outcomes

### 6.1. Study Characteristics

The major study characteristics and results for the 20 studies concerning intermediate cardiovascular outcomes in relation to acute (17 studies) (Tables 5 and 6) and/or chronic exposure (4 studies) (Table 7) are presented in Tables 5–7. The studies were conducted in underground railroad workers [43], boilermaker construction workers [44–53], highway toll collectors [54], vehicle maintenance workers [55], highway patrol officers [56], traffic policemen [57], automobile mechanics [58], and a panel of healthy volunteers exposed to welding fumes [59]. Three studies focused on identifying effect modifiers [44,50,51] (Table 5) and four re-analyzed data to identify PM_2.5_ sources and components responsible for observed associations [47,53,60,61]. In general, the studies of intermediate cardiovascular endpoints were conducted in small sample sizes (9 to 79 participants) using either short-term prospective study designs (Tables 5 and 6) with repeated measurements or cross-sectional (Table 7) designs, and were mostly male. A number of intermediate cardiovascular outcomes were studied—most commonly heart rate variability (HRV) (Table 5) and circulating markers of systemic inflammation (Table 6). Direct personal measurements of airborne PM concentrations were used to assign exposure in the boilermaker construction workers [44–53], vehicle maintenance workers [55], and highway patrol officers [23], while controlled exposures were studied in the panel of healthy volunteers exposed to welding fumes [59]. Job title was used in the other cohorts which were studied using a cross-sectional design [43,54,57,58] (Table 7). Because of the smaller number of participants, which generally makes it more feasible to collect more detailed information on a larger number of covariates, control or evaluation of important potential confounders such as smoking was conducted in all of the studies of intermediate cardiovascular outcomes, either through study design or in analysis. In addition, a number of other important potential confounders were considered in the vast majority of these studies, including co-pollutants such as CO [55,56].

### 6.2. Acute Effects

Heart rate variability (HRV), a widely-used indicator of cardiac autonomic nervous system function measured by ambulatory electrocardiogram (ECG), was measured in four cohorts (boilermakers, healthy volunteers exposed to welding fumes, vehicle maintenance workers, and highway patrol officers). HRV can be characterized using a number of indices (e.g., short duration measures such as the 5-min SDNN and long duration measures such as the SDNNi), reflecting different components of the autonomic nervous system. Decreased HRV is associated with increased risk of mortality in the general population and the development of non-fatal cardiac events [63,64]. Both the HRV indices as well as the conditions for ECG ascertainment varied between these studies (Table 5). Findings also varied by cohort.

In the boilermaker cohort, an inverse association between increasing PM_2.5_ exposure from welding fume and residual oil fly ash (ROFA) and decreasing HRV was first reported in 2001 using a repeated measures study design [52]. This study found a 2.66% (95%CI: −3.75% to −1.58%) decrease in 5-min SDNN for every 1 mg/m^3^ increase in the personal 4-hour moving average PM_2.5_ concentration, while controlling for smoking, age, and diurnal variation, suggesting an acute adverse effect of PM_2.5_ on the autonomic nervous system, a potential mechanism by which PM may be associated with adverse clinical cardiovascular events. Subsequently, consistent inverse associations between personal PM_2.5_ measurements and other measures of HRV were observed in additional boilermaker studies while controlling for smoking, age, and diurnal variation [46,48,51]. These studies strongly suggest acute adverse cardiovascular health effects of PM_2.5_ exposure from welding fumes and residual oil fly ash. Presence of co-pollutants was reported to be minimal and uncorrelated with PM_2.5_ in these cohorts, providing further support that the observed effects were due to PM_2.5_. Re-analyses of some of these studies using PM_2.5_ components showed that nighttime root mean square successive differences (rMSSD) in heart period series, a high-frequency component of HRV that reflects the relative influence of the parasympathetic nervous system, was inversely associated with manganese [47]. In an earlier study, the mean of the standard deviations of all normal to normal intervals (SDNNi) for all five minute segments of the ECG recording, an HRV measure that reflects overall contributions of the parasympathetic and sympathetic nervous system, was positively associated with lead and vanadium [53], a finding that suggests that these PM_2.5_ metal components are not the responsible constituents for observed deceases in HRV with increasing PM_2.5_ [53]. Additional studies in this cohort showed that obesity, poorer cardiovascular disease risk profile as measured by the Framingham heart score, as well poorer vascular function and greater systemic inflammation aggravate the PM_2.5_-HRV association up to four-fold [44,50,51].

In contrast to the boilermaker studies, using a repeated measures study design, associations between PM_2.5_ and a number of HRV indices immediately after a work-shift were generally null in non-smoking highway patrol officers [56], though positive associations with a number of HRV parameters were observed the morning after work (e.g., 11.7% change in next-morning SDNN per 10 μg/m^3^ increase in PM_2.5_, p = 0.006) (Table 3). Because the study participants were young and in good physical health, the authors suggested that the positive associations may reflect a healthy physiologic response to PM exposure in comparison with older and less healthy individuals and workers. The effects of co-exposures from CO, NO_2_, and relative humidity were assessed, giving minimal changes in the results, but because of the correlation with PM_2.5_ potentially confounding effects from these co-exposures could not be entirely ruled out. In subsequent analyses, PM_2.5_ associated with vehicular “speed change” (e.g., engine emissions and break wear) which include the components of copper and sulfur were associated with the increased HRV indices in the patrol officers [61]. In studies of healthy volunteers exposed to welding fumes and in vehicle maintenance workers [55,59] null associations between exposure and HRV were also observed..

Circulating markers of inflammation, coagulation, and vascular function were investigated in the cohorts of underground railways workers [43], boilermaker construction workers[49,62], healthy volunteers exposed to welding fumes [59], and highway patrol troopers [56] in relation to short-term PM exposure. The types of markers measured and their associations varied from study to study, however positive associations with inflammatory markers were more consistently observed than HRV across cohorts. For example, C-reactive protein (CRP), a commonly measured biomarker of systemic inflammation, was measured in four studies [43,49,56,59]. A positive baseline to next morning change (24 hours) in CRP (mg/L) was observed in boilermaker construction workers exposed to welding fume PM_2.5_ for approximately six hours, after adjusting for smoking and time of day (β = 0.95; 95% CI: 0.23–1.67) [49]. Similarly, in non-smoking highway patrol officers exposed to in-vehicle PM_2.5_ during a full work shift, CRP (mg/L) measured 14 hours after a shift increased 32% (p = 0.02) per 10 μg/m^3^ increase in PM_2.5_ [56]. No associations however were found in healthy volunteers exposed to welding fume using a crossover study design [59] or in underground railroad system workers with measurements taken two days apart [43]. The short duration of exposure in the healthy volunteers and/or the timing of the outcome measurements may explain the lack of effects observed in this study. An increase in absolute neutrophil counts from baseline to post-shift (× 10^3^/μl), another indicator of systemic inflammation, was also associated with increasing PM_2.5_ exposure in non-smoking boilermakers (β = 0.3; 95% CI: 0.02–0.6) [49]. In the non-smoking highway patrol officers, a 6.2% increase in percent neutrophils per 10 μg/m^3^ increase in PM_2.5_ was observed 14 hours post-shift [56]. The increase in percent neutrophils in the highway patrol officers was also linked with copper and sulfur in PM_2.5_ from vehicular “speed change”. In contrast, to these findings, no changes were observed in circulating levels of inflammatory cytokines (tumor necrosis factor [TNF]-α, interleukin-6 [IL-6], or interleukin-8 [IL-8]) in healthy volunteers exposed to welding fumes for two hours [59], which may be due to the short duration of exposure and/or timing of outcome measurements.

Soluble adhesion molecules, which relate to both inflammation and endothelial function, were also measured in relation to PM_2.5_ exposure in some studies [59,62]. Soluble inter-cellular adhesion molecule-1 (sICAM-1) was measured in boilermakers [62] and healthy volunteers exposed to welding fumes [59], but no associations with exposure were observed in either cohorts. An acute decrease in soluble vascular cell adhesion molecule-1 (sVCAM-1) however was associated with increasing PM_2.5_ exposure in boilermakers [62]. This may reflect an initial down-regulation followed by an up-regulation in VCAM-1 as explained by the authors. Von Willebrand factor (vWF), a coagulatory molecule, also related to endothelial function and inflammation, was found to increase in the highway patrol officers in relation to increasing personal PM_2.5_ exposures [56]. An acute increase in vWF across a work-shift was also observed in the cohort of boilermaker construction workers in association with personal PM_2.5_ levels [62]. Higher levels of vWF were also observed in healthy volunteers after exposure to welding fumes as compared to a control day, though this difference was only marginally statistically significant [59].

Plasminogen activator inhibitor-1 (PAI-1) and fibrinogen, which characterize a pro-thrombotic state, were measured in the underground railroad system workers, and acute increases were observed in the low-exposed workers but not the higher exposed workers [43], which could indicate a greater reactivity to occupational PM among lower exposed individuals who may have a lower baseline inflammatory state. In the healthy volunteers exposed to welding fumes, however, no differences in fibrinogen levels were observed under exposed conditions *versus* unexposed conditions [59].

In the boilermakers cohort, acute vascular effects of PM exposure were also assessed non-invasively with aortic pulse wave analysis derived from radial waveforms [45]. An acute alteration in vascular function was observed in relation to PM_2.5_ exposure as measured by the augmentation index (AIx), a correlate of arterial stiffness, while adjusting for smoking, age, and diurnal variation. Increasing AIx is an independent marker of coronary artery disease and correlates with risk of developing coronary artery disease [65,66].

### 6.3. Chronic Effects

To assess the chronic effects of exposure, four cross-sectional studies investigated the association between exposure as measured by job title and levels of circulating inflammatory markers [43], cardiovascular abnormalities [57], and measures of pre-clinical atherosclerosis [54,58] while controlling for smoking and a number of potentially important confounders (Table 7). In underground railway workers with relatively high exposure to PM_2.5_, increased baseline levels of PAI-1 and CRP were found as compared to low-exposed workers, suggesting a chronic systemic inflammatory effect of PM exposure [43]. In traffic policemen exposed to traffic-related PM, increased resting diastolic blood pressure, alterations in the ST segment of the electrical activity of the heart and arrhythmias with exercise were observed in comparison with office workers [57]. Increased homocystiene levels and decreased ocular blood flow velocity, indicators of pre-clinical atherosclerosis were observed in toll collectors *versus* non-exposed controls in Turkey, suggesting an effect of chronic PM exposure on the development of atherosclerosis [54]. Increased plasma susceptibility to oxidation in automobile mechanics as compared to white collar further supports a potential effect of chronic occupational PM exposure on pre-clinical atherosclerosis [58]. While these studies controlled for a number of potential confounders, a limitation was the lack of data on co-pollutants such as CO.

## 7. Discussion

Findings from this systematic review suggest a possible association between occupational PM exposure and IHD and stronger associations with intermediate outcomes such as heart rate variability systemic inflammation. In meta-analyses of mortality studies, risk of IHD was slightly increased, though not statistically significantly, in comparison with general populations. However, the risk of IHD among highly exposed workers compared to low exposed workers was significantly increased in pooled meta-analysis. Though adequate control for confounding factors was lacking, this finding is consistent with the fact that using internal analysis minimizes the healthy worker effect that arises when comparing relatively healthy working populations to the general population which includes unhealthy individuals unable to work. In sub-group analyses, silica-exposed cohorts in particular were found to be at increased risk of IHD mortality. No associations were found with cerebrovascular disease or overall CVD mortality. The internal analyses helped to overcome the limitations of SMR studies; however the findings remain limited because of the lack of control for smoking habits, and other potential confounders, such as age and co-pollutants, in the analyses. The review of individual morbidity studies also suggested an association with IHD as associations were observed with MI, but were also limited by lack of adequate measurement and control for co-pollutants, as exposure was primarily assessed by job title. However the findings are consistent with ambient air pollution studies which have found increases in MI with increasing particulate exposure [5,67–69].

Moreover, this review found stronger evidence of associations between acute and chronic occupational PM exposure and circulating markers of inflammation and coagulation, including CRP, neutrophils, and vWF, a plausible biological mechanism linking occupational PM exposure with IHD and MI. In addition, there was also evidence of an association between short-term occupational PM exposures and decreased HRV. All of these intermediate studies were able to control for confounding by smoking and other important risk factor either through data collection and analysis of study design. Though in the studies of chronic exposures on intermediate outcomes, in which exposure was assessed primarily by job title, lack of data on co-pollutants is of concern. The clinical significance however of short-term and even long-term effects of occupational PM exposures on intermediate pre-clinical outcomes remains to be understood and future research should focus on clarifying the clinical significance.

A number of studies in the ambient particulate air pollution literature also support a link between PM exposure and systemic inflammation [70–76], which is recognized to play an important role in the development and aggravation of IHD. The early upstream effect of PM on systemic inflammation itself may be explained by an initial localized inflammatory response in the lungs once PM is inhaled and deposited in the alveoli [77,78]. The finding that silica in particular was associated with IHD is intriguing as silica has a well-established effect on pulmonary inflammation [79].

While systemic inflammation appears to be an important intermediate outcome between exposure and adverse clinical cardiovascular outcomes, it is likely that biological mechanisms are extremely complex, involving a number of different pathways. Alterations in autonomic nervous system function have also been proposed as a biological mechanism and have been widely studied in the air pollution literature [80–82]. There is considerable evidence for a link between alterations in autonomic nervous system function and changes in particulate air pollution exposure patterns [4], and some evidence for this link for some of the types of exposures in this review, suggesting differences in the toxicity of PM by source and/or a healthy worker effect. For example, in the cohort of boilermaker construction workers, exposure to PM_2.5_ contained in welding fumes and residual oil fly ash (ROFA) was consistently associated with decreased heart rate variability (HRV), an indicator of poor cardiac autonomic nervous system function. In highway patrol troopers, positive associations were found with some HRV parameters and no associations with others. Moreover, no association between personal occupational exposure to PM_2.5_ from automobile emissions and HRV were observed in a small group of vehicle maintenance workers. Differences are likely to be due to differing types of particles as well as the timing of responses. Further research in other occupational cohorts with differing particle exposures and a panel of intermediate outcomes will help elucidate the biological mechanisms.

We acknowledge that this systematic review is not without limitations and potential biases, some of which are common to systematic reviews in general. For example, publication bias, which is the tendency for manuscripts with positive findings to be published more than those with null findings, is a potential limitation of all systematic reviews. Because of the small number of studies included in the meta-analysis portion of this review, however, it was not possible to formally assess publication bias. Given the mix of positive and null effects found in the studies included in this review, however, publication bias should not be a major concern. Of greater concern are the biases and confounding factors inherent in the individual studies. For example, uncontrolled and residual confounding by factors such as smoking and co-exposures, particularly in the mortality and morbidity studies are a major concern. Only two mortality studies controlled for smoking habits. However both these studies also controlled for potential intermediate health effects which may lie on the pathway between exposure and IHD, and thus it is possible that some of the effects of exposure on IHD were blocked. In addition, a wide range of exposure assessment methods was used in the different studies, with few directly measuring PM concentrations. Unlike ambient particulate air pollution studies, which can utilize routinely collected environmental monitoring data to study large populations with relatively similar exposures, thereby increasing statistical power; occupational cohort studies are limited to smaller populations with varying exposure levels within and between workers and workplaces, depending on factors such as tasks, workplace practices and engineering controls. Thus, while the exposure type is relatively homogenous, exposures are difficult to estimate and assign, and therefore exposure is often assigned to study participants by job title. This can lead to exposure misclassification which typically biases effect estimates towards the null. Another concern is the healthy worker effect in mortality and morbidity studies, which manifests as a protective association when disease rates in relatively healthy workers are compared with that of the general population. This can be particularly strong for symptomatic diseases such as IHD and may explain why an inverse association was observed in the styrene-exposed cohorts, which both used general populations as a reference. Finally, we could only evaluate the effect of particle type on cardiovascular-related mortality, and not non-fatal events, or intermediate events due to an inadequate number of studies for each outcome by type. Moreover, only a limited number of particle types could be evaluated (*i.e*., silica and styrene) because of the lack of studies on other particle types.

Despite these limitations, the collective evidence from a broad range of studies and cardiovascular outcomes suggests an association between occupational PM exposure and adverse cardiovascular events. Future studies on the association between occupational PM exposure and CVD should attempt to overcome the limitations noted above. Specifically, validated detailed exposure assessment methods should be utilized in future studies to reduce exposure misclassification and increase sensitivity to detect underlying associations. These methods should measure biologically relevant PM size fractions such as PM_2.5_ or UFP, in order to better characterize exposure-response relationships with cardiovascular outcomes. This can facilitate the standardization of measurements for future meta-analyses of a larger number of studies and aide in conducting risk assessments. In addition, studies that employ comparisons with an external population should attempt to control for the healthy worker effect by restricting the external population to gainfully employed individuals. Moreover, collection of data that will allow one to adjust for smoking habits and co-exposures in the population should be prioritized. Where this is not possible, efforts should be made to formally evaluate the impact of potential confounders in a sub-set of the population, or to use population-level data on smoking prevalence for instance to assess confounding. Appropriate adjustment for covariates in statistical models should consider mechanistic pathways and factors which may lie on this pathway as to not over adjust for intermediate factors, such as blood pressure or hypertension.

In conclusion, occupational PM exposure may be associated with IHD mortality and MI. There is also evidence that occupational PM exposure is associated with decreased heart rate variability, a risk factor for CVD mortality and which may be a potential mechanism of PM-associated adverse cardiovascular events and stronger evidence across study cohorts of an association with systemic inflammation, also a potential mechanism for PM-associated IHD. Though data is currently lacking to determine causality, findings from this review justify a greater recognition of the risk of both the development and aggravation of CVD from occupational exposure to PM. Future work should further assess the magnitude of the risks, including elucidation of biological mechanisms, and contribution of occupational PM exposures to the overall burden of CVD.

## Figures and Tables

**Table 1.** Epidemiologic cohort studies of particulate exposures and cardiovascular mortality included in systematic review.

**Reference**	**Country**	**Population and sample size**	**Exposure**	**Study period**	**Outcomes investigated**
Cooper, 1992 [27]	US	Iron miners and millers (taconite) employed for 3+ months in 2 companies (n = 3,431 males)	Silica	1959–1988	CVD
Reid, 1996[35]	South Africa	White gold miners who had visited the Medical Bureau for Occupational Diseases (n = 4,925 males).	Silica	1970 −1989	IHD, cerebrovascular disease
Boffetta, 1998[28]	8 European countries	Reinforced plastics industry workers (n = 14,207 males employed 1+ years)	Styrene	1940–1992	IHD
Randem, 2003[30]	Norway	Asphalt workers employed 1+ days by 13 employers (n = 8,610 males)	Asphalt fumes	1970–1996	CVD, IHD, cerebrovascular disease
Finkelstein, 2004[34]	Canada	Heavy equipment operators (n = 16,321) and other unionized workers (n = 90,675)	Diesel exhaust	1975–2000	CVD, IHD, cerebrovascular disease
Koskela, 2005[33]	Finland	Current and former workers from 6 established cohorts exposed to dust (n = 6,022)	Silica, cotton, sand, quartz, metal, solvents	1940–1992	CVD, IHD
Delzell, 2005[37]	US and Canada	Synthetic rubber industry workers from 8 plants employed for 1+ years (n = 16,579 males)	Styrene	1944–1998	IHD
Burstyn, 2005[32]	Denmark, Finland, France, Germany, Israel, Netherlands, Norway	Asphalt workers from 217 companies employed 1+ season (n = 12,367 males)	Asphalt fumes	1953–2000	CVD, IHD
Laden, 2007[31]	US	Unionized trucking industry workers employed for 1+ days (n = 54,319 males)	Diesel exhaust	1985– 2000	CVD, IHD, cerebrovascular disease
Toren, 2007[38]	Sweden	Construction workers identified via Swedish construction industry organization medical program (n = 176,309 exposed & n = 71,778 unexposed males)	Inorganic dust, fumes, diesel exhaust, asphalt fumes, metal fumes	1971–2002	IHD, cerebrovascular disease
Weiner, 2007[36]	Sweden	Miners, well borers, dressing plant workers and stone worker identified by occupational codes in the Swedish National Census of 1970 (n = 11,896 males)	Silica	1970– 1995	IHD
Zhang, 2008[29]	China	Ceramics factory workers from 3 plants (n = 4,851 males and females)	Silica	1972–2003	CVD, cerebrovascular disease

CVD: cardiovascular disease; IHD: ischemic heart disease.

**Table 2.** Findings from SMR and PMR studies of particulate exposures and cardiovascular mortality included in systematic review.

**Exposure type**	**Reference**	**External comparison group**	**Population characteristics standardized to**	**CVD** **SMR (95% CI)**	**IHD** **SMR (95% CI)**	**Cerebrovascular disease SMR (95% CI)**
Silica	Cooper, 1992 [27]	General male population	Age and calendar year	0.91 (0.83–1.00)	NA	NA
	Reid, 1996 [35]	General white male population	Age and calendar year	NA	1.24 (1.15–1.34)	1.09 (0.89–1.31)
	Weiner, 2007[36]	Employed male population	Age and calendar year	NA	1.31 (1.24–1.38)	NA
	Zhang, 2008[29]	General male and female population	Age and calendar year	1.06 (0.94–1.19)	NA	0.81 (0.71–0.93)
Styrene	Boffetta, 1998[28]	General male population	Age and calendar year	NA	0.94 (0.85–1.04)	NA
	Delzell, 2005[37]	General male population	Race, age, calendar year	NA	0.90 (0.85–0.95)	NA
Asphalt fumes	Randem, 2003[30]	General male population	Age and calendar year	0.86 (0.75–0.98)	1.14 (0.89–1.43)	0.93 (0.83–1.03)
Diesel exhaust	Laden, 2007[31]	General male population	Race, age, calendar year	0.73 (0.69–0.76)	1.41 (1.33–1.49)	0.69 (0.59–0.80)

				**PMR (95% CI)**	**PMR (95% CI)**	**PMR (95% CI)**
	Finkelstein, 2004[34]	General population	Age and calendar year	1.00 (0.90 – 1.1)	1.09 (0.96 – 1.2)	0.86 (0.60–1.2)

CVD: cardiovascular disease; IHD: ischemic heart disease; *case control analysis also conducted; *reported as an OR.

**Table 3.** Characteristics and main findings from mortality studies of occupational PM exposure and cardiovascular disease using internal analyses.

**Exposure type**	**Reference**	**Controlled confounders**	**Comparison**	**Cardiovascular disease IRR (95% CI)**	**Ischemic heart disease IRR (95% CI)**	**Cerebrovascular disease IRR (95% CI)**
Silica	Reid, 1996 [35]	Smoking, blood pressure, obesity	Unit increase in cumulative dust exposure lagged 5 yrs (mg-yr/m^3^)	NA	0.97 (0.83–1.10)	NA
	Zhang, 2008[29]	None reported	High *vs.* low cumulative exposure	0.77 (0.61–0.98)	NA	NA
Styrene	Delzell, 2005[37]	Time since hire, cumulative exposure to 1,3-butadiene	High *vs.* low cumulative exposure	NA	1.07 (0.90–1.27)	NA
			High *vs.* low lifetime average intensity	NA	1.14 (0.96–1.35)	NA
Asphalt fumes	Burstyn, 2005[32]	None	High average coal tar exposure *vs.* unexposed	1.85 (1.17–2.91)	1.64 (1.13–2.38)	NA
Silica, sand, quartz, metal, solvents	Koskela, 2005[33]	Age	High *vs.* low exposed granite workers	1.10 (0.89–1.29)	1.20 (0.94–1.58)	NA
			High *vs.* low exposed foundry workers	1.00 (0.79–1.14)	0.90 (0.71–1.16)	NA
			High *vs.* low exposed metal workers	1.20 (1.04–1.35)	1.40 (1.19–1.74)	NA
Inorganic dust, fumes, diesel exhaust, asphalt fumes, metal fumes	Toren, 2007[38]	Smoking, age, hypertension, and body mass index	Any occupational PM exposure *vs.* none	NA	1.12 (1.10–1.14)	0.97 (0.93–1.01)
			Any exposure to inorganic dust *vs.* none	NA	1.13 (1.10–1.16)	0.97 (0.9–1.02)
			Any exposure to fumes *vs.* none	NA	1.12 (1.0–1.16)	1.03 (0.95–.11)
			Any exposure to diesel exhaust *vs.* none	NA	1.18 (1.13–1.24)	1.09 (0.99–1.20)
			Any exposure to asphalt fumes *vs.* none	NA	1.12 (0.96–1.30)	1.18 (0.86–1.58)
			Any exposure to metal fumes *vs.* none	NA	1.01 (0.95–1.08)	0.92 (0.80–1.05)
Diesel exhaust particles	Finkelstein, 2004 [34]	Age	Heavy equipment operators *vs.* other workers	NA	1.23 (1.00–1.51)*	NA

* Reported as an odds ratio.

**Table 4.** Study characteristics and findings of studies on occupational particulate exposures and cardiovascular morbidity.

**Reference and country**	**Study population, size, ages, study period**	**Outcome and ascertainment**	**Exposure and assessment**	**Controlled confounders**	**Comparison**	**MI IRR (95% CI)**	**Other (95%CI)**
Cross-sectional studies							

Fleming, 2001[40] England	Employed males in the Fourth National Morbidity Survey Participants (n = 93,692 men) Ages 16–64 year 1991–1992	IHD and CVD identified from questionnaire	Traffic-related particles, assessed via self-reported job title	Smoking and age	Transport worker *vs.* general employed male population	NA	IHD prevalence RR = 0.93 (0.72–1.15)
							CVD prevalence RR = 0.99 (0. 90–1.08)
Suadicani, 2002[26] Denmark	The Copenhagen Male Study Population (n = 3,321men) Ages 53–74 year 1985–1993	MI identified from national registers of hospital admissions and death certificates	Welding and soldering fumes, assessed via questionnaire	None reported	Exposure to welding fumes several times/wk >5 yrs (yes/no)	1.1 (0.6–2.2)*	MI prevalence OR = 2.1(1.05–4.2)
					Exposure to soldering fumes several times/wk >5 yrs (yes/no)	1.8 (1.0–3.2)*	MI prevalence OR = 3.0 (1.6–5.8)

Case control studies							

Gustavsson, 1996[25] Sweden	Males in Stockholm same job for ≥5 yrs (n = 4,105 cases and 13,066 controls) Ages 30–74 year 1976–1984	1^st^ fatal and nonfatal MI identified from registers of hospital admissions and causes of death	Traffic-related particles, assessed via National Census job titles	Age, calendar year, county of residence	Bus driver *vs.* gainfully employed men	1.53 (1.15–2.05)	NA
				Smoking, overweight were indirectly evaluated	Taxi driver *vs.* gainfully employed men	1.65 (1.30–2.11)	
					Long distance lorry driver gainfully employed men	1.31 (1.05–1.64)	
					Short distance lorry gainfully employed men	1.06 (0.69–1.65)	
Gustavsson, 2001[41] Sweden	Male and female residents without history of MI (n = 1,335 cases and 1,658 controls) Ages 45–70 year 1992–1994	Fatal and non-fatal MI identified fro coronary or intensive care units and hospital discharge register	Combustion products (i.e., respirable PM), assessed via lifetime occupational history questionnaire	Smoking, age, sex, year of enrollment, hospital catchment area, alcohol, hypertension, overweight, diabetes, physical inactivity	High annual average exposure intensity (≥2.5 mg/m^3^) *vs.* unexposed	2.11 (1.23–3.60)	NA
Bigert, 2007[42] Sweden	Male residents of Stockholm (n = 22,311 cases and 131,496 controls). Ages 40–69 year 1976–1996	1^st^ MI identified from hospital discharge records	Particulate exposures among subway driver, job title determined from National Census job titles	Age and calendar year	Subway drivers *vs.* other gainfully employed men	1.06 (0.78–1.43)	NA
					Subway drivers *vs.* other manual workers	0.92 (0.6–1.25)	

* Reported as odds ratio (OR); CVD: cardiovascular disease; IHD: ischemic heart disease; MI=myocardial infarction; IRR=incidence rate ratio; NA=not applicable.

**Table 5.** Study characteristics and findings of prospective epidemiologic studies of short-term occupational particulate exposure and cardiac outcomes assessed by ambulatory ECG.

**Reference and country**	**Population and size**	**Outcomes measured**	**Exposure**	**Comparison**	**Controlled/evaluated confounders**	**Main associations with exposure**
**Short duration HRV measures obtained with resting protocol**						

Riediker, 2004 [56] US	9 non-smoking male highway patrol troopers	HR and HRV over 10 minutes, with resting protocol, before and after shift (before bed and after waking) (MCL, SDNN, PNN50, LF, HF, TP, LF/HF, ectopic beats)	In-vehicle PM_2.5_	↑ in in-vehicle work-shift PM_2.5_	Smoking, temperature, RH, work load, average speed during the shift	↑ HRV (SDNN, pNN50, HF) next morning
						↑ MCL
						↑ Ectopic beats next morning
Riediker, 2004* [61] US	9 non-smoking male highway patrol troopers	HR and HRV over 10 minutes, with resting protocol, before and after shift (before bed and after waking) (MCL, SDNN, PNN50, LF, HF, TP, LF/HF, ectopic beats)	In vehicle PM_2.5_ from “speed change” factors (Cu, S, aldehydes)	↑ in in-vehicle work-shift PM_2.5_ source factor	Smoking, CO, NO_2_, RH, stress	↑ HRV
						↑ MCL
						↑ supraventricular ectopic beats
Eninger, 2004 [55] US	5 male vehicle maintenance workers	HRV over 5 minutes with resting protocol at end of shift, (SDNN, rMSSD, TP, HF)	Vehicular PM_2.5_	↑ in TWA work-shift PM_2.5_	Smoking, age, heart rate, CO	None
Scharrer, 2007 [59] Germany	20 non-smoking male and female healthy volunteers	HRV over 10-min with resting protocol, (SDNN, TP, HF, LF)	Welding fume	Exposed condition *vs.* unexposed condition	Smoking, time of day, airway disease, CVD	None
Riediker, 2007* [60] US	9 non-smoking male highway patrol troopers	HR and HRV over 10 minutes, with resting protocol, before and after shift (before bed and after waking) (MCL, SDNN, PNN50, LF, HF, TP, LF/HF, ectopic beats)	In vehicle PM_2.5_ components	↑ in in-vehicle work-shift PM_2.5_ component	Smoking, CO, NO_2_	↑ ventricular ectopic beats with S

**Short duration HRV measures obtained from continuous ECG monitoring**						

Magari, 2001[52] US	40 male boilermakers	HRV over 24 hours, short-duration measures (5-min SDNN)	Welding fume and ROFA PM_2.5_	↑ in 4-hr moving average PM_2.5_	Smoking, age, time of day	↓ HRV (5-minute SDNN)
Chen, 2006** [51] US	10 male boilermakers	HRV over 24 hours, short-duration measures (5-min SDNN, HF)	Welding fume PM_2.5_	↑ in 4-hr moving average PM_2.5_	Framingham score, body mass index, drinking habits, smoking, time of day, eating, coffee and alcohol consumption, exercising, sleeping, heart rate	↓ HRV (5-min SDNN and HF), greater in high CVD risk individuals
		Modifiers: Coronary risk profile				
Chen, 2007** [50] US	18 male boilermakers	HRV over 24 and 48 hours, short-duration measures (5-min SDNN)	Welding fume PM_2.5_	↑ in 4-hour moving average PM_2.5_	Smoking, age, drinking, calendar year, blood pressure, obesity, time of day, eating, coffee and alcohol consumption, exercising, sleeping, workday	↓ HRV (5-min SDNN), greater in obese individuals
		Modifiers: Obesity				
Fang, 2009** [44] US	23 male boilermakers	HRV over 24 hours short duration	Welding fume PM_2.5_	↑ in 1-hour moving average PM_2.5_	Smoking, age, time of day, coffee and alcohol consumption	↓ HRV (5-min SDNN) greater in individuals with decreased vascular function and increased systemic inflammation
		Modifiers: AIx, MAP, PP, CRP				

**Intermediate and long-duration HRV measures obtained from continuous ECG monitoring**						

Cavallari, 2008[48] US	36 male boilermakers	HRV over 24 hours, intermediate duration	Welding fume and ROFA PM_2.5_	↑ in work-shift PM_2.5_	Smoking, age, non-work HRV	↓ HRV (hourly SDNNi) up to 14 hours post work
Magari, 2002* [53] US	39 male boilermakers	HRV over 24 hours, long duration measures (SDNNi)	Welding fume and ROFA PM_2.5_ metal components	↑ in work-shift PM_2.5_ metal component	Smoking, age, time of day	↑ HRV (SDNNi) associated with Pb and V
Cavallari, 2007[46] US	36 male boilermakers	HRV over 24 hours, long duration measures (24-hr, daytime, and nighttime rMSSD, SDNN, and SDNNi)	Welding fume PM_2.5_	↑ in work-shift PM_2.5_	Smoking, age, and non-work HRV	↓ HRV (24-hr, daytime, and nighttime rMSSD, SDNN, and SDNNi)
Cavallari, 2008*[47] US	26 male boilermakers	HRV over 24 hours, long duration measures (nighttime rMSSD)	Welding fume PM_2.5_ metal components	↑ in work-shift PM_2.5_ metal component	Smoking, age, non-work HRV	↓ HRV as measured by nighttime rMSSD associated with Mn

*CVD:* cardiovascular disease; *ECG:* electrocardiogram; *HF:* high frequency (HRV index that reflects parasympathetic autonomic nervous system control); *HRV:* heart rate variability; *LF:* low frequency (; *MAP:* mean arterial pressure; *MCL:* mean cycle length; PM_2.5_: particulate matter with a mass mean aerodynamic diameter ≤ 2.5 μm; pNN50: Percentage of differences between adjacent NN intervals that are >50 msec; *PP:* pulse pressure; *rMSSD:* root mean square of successive differences (HRV index that reflects relative influence of parasympathetic nervous system activity); *ROFA:* residual oil fly ash; *SBP:* systolic blood pressure; *SDNN*: standard deviation of normal of normal beats (HRV index that reflects total HRV, sympathetic and parasympathetic components, over a specified time); *SDNNi:* SDNN index; *TP:* total power (HRV index that reflects total HRV over a specified time).

**Table 6.** Study characteristics and findings of prospective epidemiologic studies on short-term occupational particulate exposures and inflammation, coagulation, and vascular function.

**Reference and country**	**Population and size**	**Outcomes measured**	**Exposure**	**Comparison**	**Controlled confounders**	**Main associations with exposure**
Riediker, 2004 [56] US	9 non-smoking male highway patrol troopers	**Inflammation:** circulating CRP, lymphocytes, neutrophils, vWF, RBC indices	In vehicle PM_2.5_	↑ in PM_2.5_	Smoking, temperature, RH, workload, and average speed	↑ in RBC indices, neutrophils, CRP, and vWF ↓ lymphocytes
Riediker, 2004* [61] US	9 non-smoking male highway patrol troopers	**Inflammation:** circulating CRP, lymphocytes, neutrophils, vWF, RBC indices	In vehicle PM_2.5_ from “speed change”	↑ in PM_2.5_ source factor	Smoking, CO, NO_2_, RH, stress	↑ in neutrophils, RBC volume MCV, and vWF ↓ lymphocytes
Kim, 2005 [49] US	37 male boilermakers	**Inflammation:** circulating CRP, fibrinogen, neutrophils, WBCs	Welding fume PM_2.5_	↑ in work-shift TWA PM_2.5_	Smoking, age, time of day	↑ absolute neutrophil counts in non-smokers ↑ CRP levels in non-smokers and smokers
Scharrer, 2007[59] Germany	20 non-smoking male and female healthy volunteers	**Inflammation:** circulating antithrombin III, ristocetin cofactor,CRP, ENA78, ET-, factor VIII, fibrinogen, IL-6, IL-8, ristocetin, sICAM-1, TNF-a vWF	Welding fume	Exposed condition *vs.* unexposed condition	Smoking, time of day, airway disease, CVD	↓ ET-1
Riediker, 2007[60]* US	9 non-smoking male highway patrol troopers	**Inflammation:** circulating IL-6 PAI-1 vWF, WBCs	In-vehicle PM_2.5_ components	↑ in in-vehicle PM_2.5_ component	Smoking, CO, NO_2_	↑ vWF and uric acid with Ca ↑ WBCs and IL-6 with Cr ↑ vWF, MCL ↓ PAI-1 with Cu
Bigert, 2008 [43] Sweden	79 non-smoking male and female Stockholm underground workers	**Inflammation and coagulation:** circulating PAI-1	Underground railroad PM_2.5_	48 hour change from start of work-shift in low, medium, and high exposure groups	Smoking, anti-coagulation medication	↑ PAI-1 and fibrinogen after 2 days of work in lower exposed workers
Fang, 2009[62] US	26 male boilermakers	**Inflammation, coagulation, and endothelial function:** circulating sICAM-1, sVCAM-1, and vWF,	Welding fume PM_2.5_	↑ in TWA work-shift PM_2.5_	Smoking, age, time of day	↑ vWF post-shift ↓ sVCAM-1 post-shift and next morning
Fang, 2008[45] US	26 male boilermakers	**Vascular function:** augmentation index	Welding fume PM_2.5_	↑ in TWA work-shift PM_2.5_	Smoking, age, time of day	↑ augmentation index post-shift

*CRP:* C-reactive protein; *ENA78:* Epithelial cell-derived neutrophil-activating peptide 78; *ET-1:* endothelin-1; *IL-6:* interleukin 6; *IL-8;* interleukin-8; *PAI-1:* plasminogen activator inhibitor-1; PM_2.5_: particulate matter with a mass mean aerodynamic diameter ≤ 2.5 μm; *RH:* relative humidity; *sICAM-1:* soluble inter-cellular adhesion molecule-1; *sVCAM-1:* soluble vascular cell adhesion molecule-1; *TNF-a:* tumor necrosis factor alpha; *vWF:* von Willebrand factor; *WBC:* white blood cell.

**Table 7.** Study characteristics and findings of cross-sectional epidemiologic studies of chronic occupational particulate exposure and intermediate outcomes.

**Reference and country**	**Population and size**	**Outcomes measured**	**Exposure**	**Comparison**	**Controlled confounders**	**Main associations with exposure**
Sharman, 2002 [58] Australia	16 automobile mechanics and 13 white collar workers	**Pre-clinical atherosclerosis:** Plasma susceptibility to oxidation (lag time, change in absorbance, slope of linear time trend)	Automobile pollutants	Automobile mechanics *vs.* white collar workers	Smoking, age, CVD, hyperlipidemia, hypertension, obesity, antioxidants, aerobic capacity	↑ change in absorbance at 234 nm ↑ linear slope of oxidation curve
Volpino, 2004 [57] Italy	68 male traffic policemen and 62 office workers	**Vascular function:** diastolic and systolic blood pressures **Cardiac function and abnormalities:** heart rate, ECG abnormalities (ST segment alterations, arrhythmia)	Traffic-related air pollution	Traffic policemen *vs.* office workers	Smoking, age, residence, duration of employment	↑ diastolic blood pressure at rest and with exercise ↑ ST segment alterations with exercise ↑ Arrhythmia with exercise
Memisogullari, 2007[54] Turkey	22 male non-smoking highway toll collectors and 24 controls	**Pre-clinical atherosclerosis:** circulating homocysteine levels and ocular blood flow velocities (measured by Doppler ultrasonography)	Motor exhaust particles	Toll collectors *vs.* controls	Smoking, cholesterol, glucose, acute and chronic inflammatory disease, medications and vitamins, creatinine, caffeine	↑ homocystiene levels ↑ OA RI ↓ ocular blood velocity
Bigert, 2008 [43] Sweden	79 non-smoking male and female Stockholm underground workers	**Inflammation and coagulation:** circulating PAI-1, CRP, factor VII, IL-6, fibrinogen, vWF	Underground railroad PM_2.5_	High *vs.* medium and low exposed workers	Smoking, BMI, and anti-coagulation medication	↑ PAI-1 and CRP in high exposed workers

*CRP:* C-reactive protein; *CVD:* cardiovascular disease; *OA RI:* resistivity index of the ophthalmic artery, an indirect measure of arterial resistance and the atherosclerotic process; *PAI-1:* plasminogen activator inhibitor-1; PM_2.5_: particulate matter with a mass mean aerodynamic diameter ≤ 2.5 μm.

## Appendix

### Search Terms

(Crystalline Silica OR Diesel Exhaust Emissions OR Engine Emissions OR Styrene OR Metal Fumes OR Welding Fumes OR Asphalt) AND (Cardiovascular Disease[32] OR Mortality OR Myocardial Infarction OR Peripheral Arterial Disease OR Peripheral Vascular Disease OR Hypertension OR Blood pressure OR Systolic OR Diastolic OR Atherosclerosis OR Arteriosclerosis OR Electrocardiography OR Heart Rate OR Ventricular Hypertrophy OR Heart Failure OR Ischemic Heart Disease) AND (Job* OR Work* OR Occupation*).

**Table A.** Additional characteristics of epidemiologic studies of occupational PM exposure and fatal cardiovascular disease included in review.

	**Retrospective cohort studies**											**PMS**
	Cooper et al., 1992	Reid et al. 1996	Bofetta et al. 1998	Randem et al. 2003	Koskela et al. 2005	Delzell et al. 2005	Bustyn et al. 2005	Laden et al. 2007	Toren et al. 2007	Weiner et al. 2007	Zhang et al. 2008	Finkelstein et al. 2004
**Exposure assessment**												
Estimated individual-level PM exposure (e.g. based on job title)	N	Y	N	Y	Y	Y	Y	N	Y	N	Y	N
Estimates of PM concentrations presented	N	Y	N	N	N	Y	Y	N	N	N	Y	N
**Outcome assessment**												
Vital status determined for ≥95% of the population	U	Y	Y	U	U	Y	Y	U	Y	U	N	U
Ascertainment of vital status based on national/regional registry(s)	Y	Y	U	Y	Y	Y	U	Y	Y	Y	N	Y
Cause of death based on death certificate	Y	Y	U	Y	Y	Y	U	Y	Y	Y	N	Y
**Statistical Analysis**												
External comparison presented	Y	Y	Y	Y	N	Y	N	Y	N	Y	Y	Y
Analysis minimized confounding by HWE	N	Y	Y	N	Y	Y	Y	N	Y	Y	Y	Y
External comparison restricted to working population	N	N	N	N	--	N	--	N	--	Y	N	N
Internal comparisons presented	N	Y	Y	N	Y	Y	Y	N	Y	N	Y	Y
Exposure-response relationship presented for CVD	N	N	N	Y	N	Y	Y	N	N	N	Y	N
Authors adjusted for smoking in internal comparisons	N	Y	N	N	Y	N	N	N	Y	N	N	N
If smoking not adjusted for, authors evaluated potential impact	N	--	N	Y	--	N	Y	Y	--	N	N	N
Authors presented a case control analysis	N	Y	N	N	N	N	N	N	N	N	N	Y

--= not applicable; U=unknown
